# A Nomogram Integrating CD169%, Neutrophil CD64 Index, and C-Reactive Protein for Differential Diagnosis of Mixed Respiratory Tract Infections

**DOI:** 10.3390/diagnostics16152416

**Published:** 2026-07-31

**Authors:** Yiling Fan, Lei Zhang, Jinyan Zhao, Xia Peng, Juan Wang, Lihui Lin

**Affiliations:** 1 Department of Laboratory Medicine, Shanghai Clinical Research and Trial Center, ShanghaiTech University, Shanghai 201210, China; fanttxk@163.com; 2 College of Medical Technology, Shanghai University of Medicine and Health Sciences, Shanghai 200237, China; 3 Department of Laboratory Medicine, Shanghai General Hospital, Shanghai Jiao Tong University School of Medicine, Shanghai 200080, China; 13801885102@163.com (L.Z.); jingy1213@163.com (J.Z.); pengxiajiao1@163.com (X.P.)

**Keywords:** CD169, neutrophil CD64, C-reactive protein, mixed infection, respiratory tract infection, nomogram, flow cytometry

## Abstract

**Background**: Early discrimination between viral, bacterial, and mixed respiratory tract infections remains challenging in clinical practice. Conventional microbiological methods may be limited by turnaround time and specimen-related factors, whereas routine inflammatory markers lack sufficient specificity for etiological classification. We investigated whether flow cytometry-derived immune markers combined with C-reactive protein (CRP) could improve early infection stratification. **Methods**: In this prospective single-center study, adult patients with acute respiratory tract infection and healthy controls were enrolled between 29 July 2025 and 31 January 2026. Peripheral blood CD169 positivity on monocytes (CD169%), neutrophil CD64 index (nCD64 index), monocyte HLA-DR positivity (HLA-DR%), and CRP were measured and compared across viral infection (influenza A, influenza B, or COVID-19), bacterial infection, mixed infection, and healthy control groups. Logistic regression models were developed for three diagnostic tasks: viral versus bacterial infection, mixed versus bacterial infection, and mixed versus viral infection. Model performance was evaluated using receiver operating characteristic (ROC) analysis, DeLong testing, calibration, decision curve analysis (DCA), and bootstrap internal validation. Nomograms were constructed from the final models. **Results**: A total of 216 infected patients were included: 112 with viral infection, 67 with bacterial infection, and 37 with mixed infection; 50 healthy individuals served as controls. CD169% was increased in viral and mixed infection, whereas the nCD64 index was increased in bacterial and mixed infection. HLA-DR% was lower in bacterial infection. For differentiating viral from bacterial infection, the optimized three-marker model combining CD169%, nCD64 index, and CRP achieved an area under the ROC curve (AUC) of 0.8947 (95% CI: 0.8463–0.9432), with a sensitivity of 81.25% and a specificity of 80.70%. In mixed infection, CD169% was the main discriminator versus bacterial infection, while the nCD64 index and CRP were more informative versus viral infection. The combined model yielded an AUC of 0.7618 (95% CI: 0.6485–0.8751) for mixed versus bacterial infection and 0.8406 (95% CI: 0.7594–0.9218) for mixed versus viral infection. Longitudinal analyses showed that CD169% declined during recovery from viral infection, whereas the nCD64 index and CRP decreased after treatment in bacterial infection; all three markers declined in mixed infection. **Conclusions**: CD169%, the nCD64 index, and CRP reflect complementary dimensions of the host response to acute infection. Their combined assessment showed good performance for differentiating viral from bacterial infection and provided clinically relevant information in mixed infection. This host-response-based nomogram may serve as a practical adjunct for early etiological stratification and mixed-infection risk assessment.

## 1. Introduction

Acute respiratory tract infection is a common reason for emergency evaluation, yet early etiological classification remains difficult in routine practice. Distinguishing viral, bacterial, and mixed infections at presentation is important for selecting appropriate anti-infective therapy and avoiding unnecessary antibiotic use [1]. In the early stage of illness, however, symptoms, physical findings, and routine laboratory results often overlap across pathogens, limiting their value for bedside etiological judgment [2,3]. Conventional pathogen-directed methods, including microbial culture, nucleic acid amplification, and antigen testing, remain essential for diagnosis, but each has practical limitations [4,5]. Culture is time-consuming and may be affected by prior antimicrobial exposure. Molecular and antigen-based assays depend on specimen quality, sampling timing, pathogen load, and the range of pathogens included in the testing panel. In many acute-care settings, these methods are not available quickly enough to guide initial treatment decisions [6,7]. Common inflammatory markers such as C-reactive protein (CRP), procalcitonin, and leukocyte count are readily available, but they lack sufficient specificity for confident discrimination between viral and bacterial infection and may be influenced by non-infectious inflammatory conditions [8,9,10]. Therefore, host-response markers that become abnormal early in infection may provide useful adjunctive information before microbiological results are available [11].

Flow cytometry-based immune markers have emerged as promising tools for early infection assessment because they reflect cellular immune activation rather than downstream systemic inflammation alone [12,13]. Monocyte CD169, also known as Siglec-1, is induced by type I interferon signaling and is typically upregulated during viral infection. Monocyte CD169 positivity (CD169%) has therefore been proposed as a marker of virus-associated immune activation [14,15,16,17]. Neutrophil CD64 (FcγRI) is rapidly upregulated in bacterial infection, systemic inflammation, and sepsis, and the neutrophil CD64 index (nCD64 index) has been used as an indicator of bacterial inflammation and disease severity [12,18,19]. Monocyte HLA-DR expression reflects antigen-presenting capacity and overall immune competence; reduced monocyte HLA-DR positivity (HLA-DR%) has been associated with immune suppression, severe infection, and poor clinical outcome [20,21]. Unlike routine serum markers, flow cytometry can evaluate several immune-response axes in the same blood sample, including interferon-driven monocyte activation, neutrophil activation, and monocyte antigen-presenting status [12,13].

In practice, however, no single biomarker performs well across the full spectrum of acute infection. This limitation is particularly relevant to the diagnosis of mixed infection, where viral and bacterial responses may coexist and blur the usual immunophenotypic pattern [22]. A single marker may therefore reflect only part of the underlying immune response. Combining cellular immune markers with routine inflammatory tests may improve early discrimination between infection types, especially when mixed infection is suspected [12,17]. A multivariable model may also facilitate clinical application by translating complex biomarker information into individualized risk estimates [17].

In this prospective study, we enrolled patients with acute respiratory tract infection and healthy controls to examine how CD169%, the nCD64 index, HLA-DR%, and CRP varied across infection types and how well they distinguished them. We first assessed the performance of each biomarker for separating viral from bacterial infection and then tested several combined models to identify a practical diagnostic panel. Because mixed infection is particularly difficult to recognize early, we also evaluated whether combined models could distinguish it from isolated bacterial or viral infection. In a subset of patients, we further examined biomarker changes before and after treatment to explore whether they tracked with clinical recovery [17].

## 2. Materials and Methods

### 2.1. Study Population and Ethics

This prospective study was conducted at Shanghai General Hospital between 29 July 2025 and 31 January 2026. The study protocol was approved by the Ethics Committee of Shanghai General Hospital (approval No. 2025KS401; approval date: 28 July 2025). All specimens were residual samples obtained after routine clinical testing. Because only de-identified leftover clinical specimens were used and no additional intervention was performed, the requirement for written informed consent was waived by the Ethics Committee. The study was conducted in accordance with the Declaration of Helsinki.

Consecutive adult patients presenting to the emergency department at their first visit for the current episode of suspected acute respiratory infection were screened for eligibility. Inclusion criteria were: (1) age ≥ 18 years; (2) body temperature ≥ 38.0 °C and/or respiratory symptoms such as cough, dyspnea, or sputum production; and (3) availability of residual EDTA-anticoagulated whole-blood specimens suitable for flow cytometric analysis. Exclusion criteria were: (1) pregnancy or lactation; (2) active malignancy or chemotherapy/radiotherapy within the preceding 3 months; (3) severe hepatic or renal dysfunction; (4) use of immunosuppressive agents within 4 weeks before enrollment; and (5) known autoimmune disease.

Healthy controls were recruited from individuals undergoing routine health examinations. At enrollment, they had no clinical evidence of infectious or inflammatory disease, and their routine laboratory test results were within the corresponding reference ranges.

### 2.2. Etiological Classification and Adjudication

Etiological classification was established using a composite clinical reference standard. The attending emergency physician made an initial diagnosis based on the clinical presentation, routine laboratory findings, microbiological testing, and imaging results. A second study physician subsequently reviewed all available source data and independently adjudicated each case as viral, bacterial, or viral–bacterial mixed infection. For hospitalized patients, adjudication incorporated all information available through discharge, including additional microbiological, laboratory, and imaging findings, treatment, clinical course, and discharge diagnosis. For patients discharged directly from the emergency department, classification was based primarily on pathogen-specific testing performed at the index visit together with a compatible clinical presentation, as systematic longitudinal follow-up was not available.

Viral infection, defined in this study as infection with influenza A virus, influenza B virus, or SARS-CoV-2, required a compatible acute respiratory syndrome and virus-specific laboratory evidence. Influenza A or B infection required positive results for both the corresponding virus-specific IgM antibody and viral antigen, whereas SARS-CoV-2 infection required positive results for both SARS-CoV-2-specific IgM antibody and nucleic acid testing. Bacterial infection required a positive bacterial culture, with the identified organism considered consistent with the site of infection and the clinical syndrome. Culture results were reviewed in conjunction with specimen type and quality, clinical findings, and imaging results to exclude colonization and contamination. Viral–bacterial mixed infection was defined as fulfillment of both the viral and bacterial infection criteria during the same illness episode.

Disagreements between the two physicians were resolved through joint review of the source data and consensus. Consensus was used only to resolve differences in the interpretation of adequate evidence and not to classify cases with insufficient or conflicting etiological evidence. Cases that could not be reliably classified after review of all available clinical, microbiological, imaging, and longitudinal data were designated as having an indeterminate etiology and excluded from the etiological comparisons and diagnostic-performance analyses. The primary reasons for indeterminate classification were insufficient pathogen-specific evidence, insufficient clinical information, or discordance between microbiological findings and the clinical syndrome. Each case was assigned one primary reason for nonclassification.

**Figure 1:**
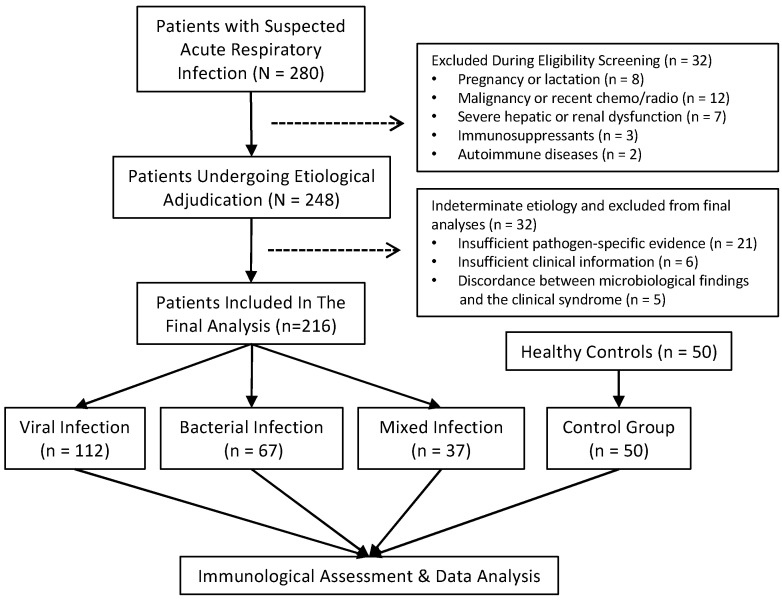
Flow diagram of patient screening, etiological adjudication, and final classification. Of the 280 patients with suspected acute respiratory tract infection who were screened, 32 were excluded during eligibility assessment according to the predefined exclusion criteria. The remaining 248 patients underwent etiological adjudication, of whom 32 were classified as having an indeterminate etiology and excluded from the final analyses. Consequently, 216 patients were included in the final infection cohort. Based on microbiological findings and comprehensive clinical assessment, infected patients were classified into the viral infection group (*n* = 112), bacterial infection group (*n* = 67), and mixed infection group (*n* = 37). In addition, 50 healthy individuals without evidence of infection or inflammatory disease were enrolled as healthy controls. All participants underwent flow cytometric analysis and statistical evaluation.

Both physicians were blinded to CD169%, HLA-DR%, and the neutrophil CD64 index throughout the initial assessment, independent adjudication, and consensus process. CRP was available as part of routine clinical care but was not used as a standalone criterion for etiological classification. The classification workflow is presented in Figure 1, and the detected pathogens and pathogen combinations are summarized in Supplementary Table S1.

### 2.3. Sample Collection and Routine Laboratory Testing

Residual EDTA-anticoagulated venous whole-blood specimens collected for routine complete blood count testing at the index emergency department visit were obtained from all participants prior to the initiation of any treatment. Routine hematological and inflammatory parameters were measured using a Mindray BC-7500CS automated hematology analyzer (Mindray, Shenzhen, China), including white blood cell count (WBC), neutrophil percentage (NEU%), lymphocyte percentage (LYM%), monocyte percentage (MO%), and CRP.

For flow cytometric analysis, all specimens were processed within 2 h of collection. All antibodies were obtained from BD Biosciences (San Jose, CA, USA), as detailed in Table 1. The antibody cocktail was freshly prepared immediately before each staining run, protected from light, and used on the same day. Briefly, 100 μL of whole blood was incubated with 65 μL of a freshly prepared antibody cocktail for 20 min at room temperature in the dark. Subsequently, 2 mL of BD FACS lysing solution (BD Biosciences, San Jose, CA, USA) was added, and erythrocytes were lysed for 10 min at room temperature. After centrifugation at 300× *g* for 5 min, the cell pellet was resuspended and immediately acquired using a BD FACSLyric multicolor flow cytometer (BD Biosciences, San Jose, CA, USA). Instrument setup, fluorescence compensation, and daily quality control were performed according to the manufacturer’s recommendations before sample acquisition.

### 2.4. Flow Cytometric Gating Strategy and Quantification of Immune Markers

**Figure 2:**
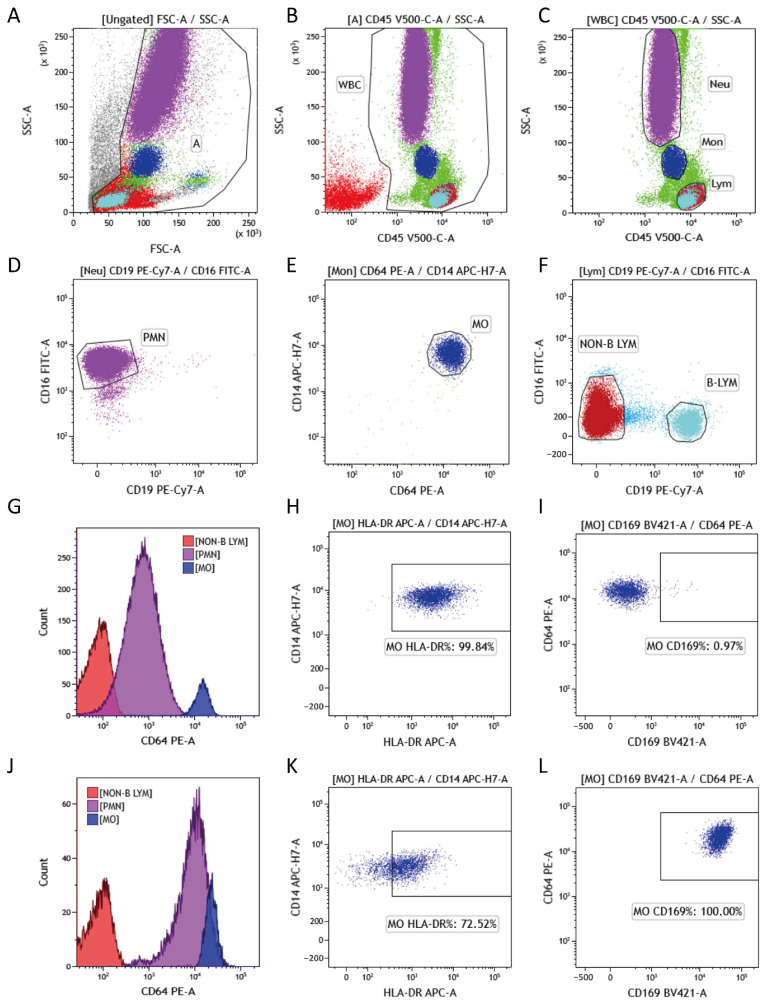
Flow cytometric gating strategy and representative expression patterns of CD169, HLA-DR, and CD64. (**A**) Cell debris and abnormal events were excluded according to forward scatter and side scatter characteristics. (**B**) CD45-positive events were gated as total leukocytes. (**C**) Neutrophils, monocytes, and lymphocytes were identified based on CD45 expression intensity and side scatter properties. (**D**) Neutrophils were further identified as CD16-positive and CD19-negative cells within the granulocyte gate (purple). (**E**) Monocytes were identified as CD14-positive cells within the monocyte gate (blue). (**F**) B lymphocytes (cyan) were identified as CD19-positive cells, whereas non-B lymphocytes (red) were defined as CD19-negative cells within the lymphocyte gate. (**G**,**J**) Representative histograms showing CD64 expression in neutrophils, monocytes, and non-B lymphocytes. (**H**,**K**) Representative dot plots showing monocyte HLA-DR expression. (**I**,**L**) Representative dot plots showing monocyte CD169 expression. Representative cases showed increased monocyte CD169 expression in viral infection, increased neutrophil CD64 expression in bacterial infection, and reduced monocyte HLA-DR expression in bacterial and mixed infection. FSC, forward scatter; SSC, side scatter; MFI, mean fluorescence intensity.

Flow cytometric data were analyzed using Kaluza software 2.4 (Beckman Coulter, Brea, CA, USA). The gating strategy is shown in Figure 2A–F. Debris and abnormal events were first excluded using forward scatter (FSC) and side scatter (SSC) properties. CD45-positive events were then selected to identify total leukocytes. Lymphocytes, monocytes, and granulocytes were distinguished based on CD45 intensity and SSC characteristics. Within the granulocyte gate, CD16-positive cells were defined as neutrophils. CD14-positive cells with high CD64 expression were identified as monocytes. Non-B lymphocytes were defined by excluding CD19-positive B cells from the lymphocyte population.

The following parameters were quantified: within the gated monocyte population, CD169% and HLA-DR% were defined as the percentages of monocytes positive for CD169 and HLA-DR, respectively; the mean fluorescence intensity (MFI) of CD64 on neutrophils, monocytes, and non-B lymphocytes were also recorded. Positivity thresholds for CD169 and HLA-DR were established using fluorescence-minus-one controls, internal negative cell populations, and healthy control samples. A uniform gating strategy was applied across all samples. The nCD64 index was calculated as follows [12,23]: nCD64 index = (neutrophil CD64 MFI/non-B lymphocyte CD64 MFI) ÷ (monocyte CD64 MFI/neutrophil CD64 MFI).

### 2.5. Development of Diagnostic Models

To assess the discriminatory performance of individual biomarkers and combined biomarker models, logistic regression models were constructed for three predefined diagnostic tasks: (1) viral infection versus bacterial infection; (2) mixed infection versus bacterial infection; and (3) mixed infection versus viral infection.

For the viral versus bacterial analysis, viral infection was defined as the positive outcome and bacterial infection as the negative outcome. Candidate predictors included CD169%, nCD64 index, HLA-DR%, and CRP. Univariable logistic regression was first performed for each biomarker. Variables showing statistical significance or considered biologically relevant were then entered into multivariable logistic regression models. Three combined models were evaluated: a three-marker flow cytometry model incorporating CD169%, nCD64 index, and HLA-DR%; a four-marker model incorporating CD169%, nCD64 index, HLA-DR%, and CRP; and an optimized three-marker model incorporating CD169%, nCD64 index, and CRP. The final model was selected based on parsimony, Akaike information criterion (AIC), regression coefficients, and overall diagnostic performance.

For mixed-infection analyses, mixed infection was defined as the positive outcome, with bacterial infection or viral infection defined as the negative outcome according to the comparison. The combined model for mixed infection included CD169%, nCD64 index, and CRP. Analyses of combined models were conducted using complete cases only; missing biomarker values were not imputed.

### 2.6. Statistical Analysis

Statistical analyses and graphical visualization were performed using R version 4.5.2 (R Foundation for Statistical Computing, Vienna, Austria) and GraphPad Prism version 10.4.1 (GraphPad Software, Boston, MA, USA).

Normality of continuous variables was assessed using the Shapiro–Wilk test. Because most continuous variables were non-normally distributed, data are presented as medians with interquartile ranges (IQRs). Comparisons among multiple groups were performed using the Kruskal–Wallis test followed by Dunn’s post hoc test. Categorical variables are expressed as counts and percentages and were compared using the chi-square test or Fisher’s exact test, as appropriate.

Univariable and multivariable logistic regression analyses were used to assess associations between biomarkers and infection categories. Regression coefficients (β), odds ratios (ORs), 95% confidence intervals (CIs), and *p* values were reported. Receiver operating characteristic (ROC) curve analysis was performed to evaluate diagnostic performance, and the area under the ROC curve (AUC), 95% CI, sensitivity, specificity, and Youden index were calculated. The optimal cutoff was determined by maximizing the Youden index. For logistic regression models, the cutoff was defined by the predicted probability threshold for the positive outcome. Pairwise comparisons of AUCs were performed using the DeLong test. Decision curve analysis (DCA) was used to evaluate clinical net benefit across a range of threshold probabilities. Calibration curves were generated to assess agreement between predicted and observed outcomes. Internal validation was performed using 1000 bootstrap resamples. Nomograms were constructed based on the final logistic regression models.

For longitudinal analyses, paired pre- and post-treatment data were analyzed using the paired Wilcoxon signed-rank test. All tests were two-sided, and *p* < 0.05 was considered statistically significant.

## 3. Results

### 3.1. Study Population and Baseline Characteristics

A total of 280 patients were initially screened, of whom 32 were excluded because they did not meet the predefined eligibility criteria. The remaining 248 patients underwent etiological adjudication. Among them, 32 patients were classified as having an indeterminate etiology and excluded from the final etiological and diagnostic-performance analyses. The primary reasons for indeterminate classification were insufficient pathogen-specific evidence (*n* = 21), insufficient clinical information (*n* = 6), and discordance between the microbiological findings and the clinical syndrome (*n* = 5). The remaining 216 patients were included in the final analysis and classified as having viral infection (*n* = 112), bacterial infection (*n* = 67), or viral-bacterial mixed infection (*n* = 37). Of the 216 patients included in the final analysis, 155 (71.8%) were hospitalized after the index emergency department visit, whereas 61 (28.2%) were discharged directly from the emergency department. The hospitalization rates were 60.7% (68/112) in the viral group, 86.6% (58/67) in the bacterial group, and 78.4% (29/37) in the mixed-infection group (Supplementary Table S2). The viral group included SARS-CoV-2, influenza A virus, influenza B virus, and combinations of these viruses. The bacterial pathogens included *Klebsiella pneumoniae*, *Staphylococcus aureus*, *Escherichia coli*, *Acinetobacter baumannii*, *Pseudomonas aeruginosa*, and *Stenotrophomonas maltophilia*. Patients in the mixed-infection group had concurrent virological and bacteriological evidence during the same illness episode (Supplementary Table S1). Fifty healthy individuals were enrolled as controls during the same period. Baseline characteristics and routine laboratory findings are summarized in Table 2.

Sex distribution was comparable across groups, whereas age differed significantly and was considered a potential confounder. Routine laboratory indicators, including WBC, NEU%, LYM%, MO%, and CRP, also differed significantly among the four groups. Patients with bacterial infection and mixed infection tended to have higher NEU% and CRP levels and lower LYM%, whereas changes in conventional inflammatory parameters were less pronounced in viral infection. The distributions of routine hematological parameters and CRP are shown in Supplementary Figure S1.

### 3.2. Flow Cytometric Immune Profiles

Multiparameter flow cytometry was used to quantify CD169%, HLA-DR%, and the nCD64 index in peripheral blood. Representative gating strategies and expression profiles are shown in Figure 2G–L. In healthy controls, monocyte CD169 expression was low, neutrophil CD64 remained at basal levels, and monocyte HLA-DR expression was relatively high. In viral infection, monocyte CD169 expression was markedly increased, consistent with activation of a virus-associated interferon response. In bacterial infection, neutrophil CD64 expression was elevated, indicating enhanced neutrophil activation. Decreased monocyte HLA-DR expression was observed in some patients with bacterial and mixed infection, suggesting impaired antigen-presenting capacity or infection-associated immune dysregulation.

### 3.3. Differences in Flow Cytometry-Derived Immune Markers Across Infection Types

**Figure 3:**
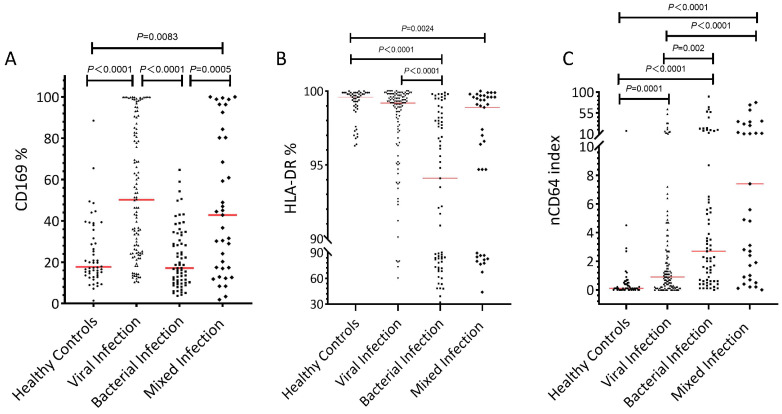
Distribution of flow cytometric immune markers among healthy controls and patients with different infection types. CD169%, HLA-DR%, and the nCD64 index were compared among healthy controls (*n* = 50), patients with viral infection (*n* = 112), patients with bacterial infection (*n* = 67), and patients with mixed infection (*n* = 37). (**A**) CD169%. (**B**) HLA-DR%. (**C**) nCD64 index. Each dot represents one participant, and horizontal lines indicate medians. Between-group comparisons were performed using the Kruskal–Wallis test followed by Dunn’s multiple-comparison test. Only statistically significant pairwise comparisons are shown. nCD64 index: neutrophil CD64 index.

The distributions of flow cytometry-derived immune markers across infection groups are shown in Figure 3. CD169% was higher in both the viral and mixed infection groups than in healthy controls and the bacterial infection group. In contrast, the nCD64 index was increased in bacterial and mixed infection and was higher than in healthy controls and viral infection. HLA-DR% was reduced in bacterial infection compared with the other groups and was also lower in mixed infection than in healthy controls. Overall, the marker profiles were consistent with distinct but partially overlapping immune patterns: viral infection was characterized mainly by increased CD169%, bacterial infection by an elevated nCD64 index with reduced HLA-DR%, and mixed infection by simultaneous elevation of CD169% and the nCD64 index with relative reduction in HLA-DR%.

### 3.4. Diagnostic Models for Discriminating Viral from Bacterial Infection

To identify an optimal biomarker combination for differentiating viral from bacterial infection, we first performed univariable logistic regression analyses using CD169%, nCD64 index, HLA-DR%, and CRP. Viral infection was defined as the positive outcome and bacterial infection as the negative outcome. All four biomarkers were significantly associated with infection category (all *p* < 0.05; Table 3). Higher CD169% and HLA-DR% were associated with a greater probability of viral infection, whereas higher nCD64 index and CRP were associated with bacterial infection.

We then constructed multivariable logistic regression models to compare different biomarker combinations. The initial three-marker flow cytometry model included CD169%, nCD64 index, and HLA-DR%. The four-marker model additionally incorporated CRP. The optimized three-marker model included CD169%, nCD64 index, and CRP. In the initial three-marker model, all three flow cytometry-derived markers were independently associated with viral infection. However, after CRP was added, the effect of HLA-DR% was markedly attenuated and was no longer statistically significant (OR = 1.001, 95% CI: 0.9553–1.0580, *p* = 0.9678), suggesting that HLA-DR% added little incremental diagnostic information beyond CD169%, nCD64 index, and CRP. The optimized three-marker model was therefore selected for subsequent analyses. In this model, CD169% remained a positive predictor of viral infection (OR = 1.0581, 95% CI: 1.0327–1.0840, *p* < 0.0001), whereas CRP was a negative predictor (OR = 0.9681, 95% CI: 0.9538–0.9827, *p* < 0.0001). The nCD64 index showed a negative association with viral infection and approached statistical significance (OR = 0.9611, 95% CI: 0.9228–1.0010, *p* = 0.0559) (Supplementary Table S3).

**Figure 4:**
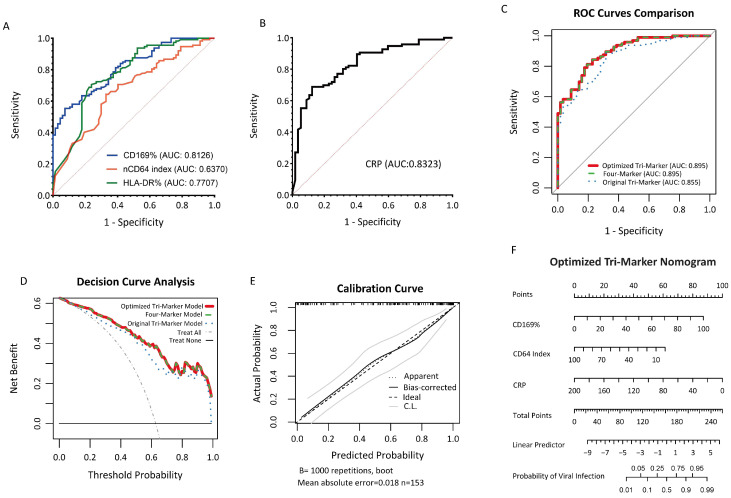
Diagnostic performance, clinical utility, calibration, and nomogram for differentiating viral infection from bacterial infection. Viral infection was coded as the positive outcome. (**A**) Receiver operating characteristic curves of individual flow cytometric markers, including CD169%, HLA-DR%, and the nCD64 index. (**B**) Receiver operating characteristic curve of CRP. (**C**) Comparison of combined diagnostic models, including the initial three-marker flow cytometric model, the four-marker model, and the optimized three-marker model. The initial three-marker flow cytometric model included CD169%, HLA-DR%, and the nCD64 index; the four-marker model included CD169%, HLA-DR%, nCD64 index, and CRP; and the optimized three-marker model included CD169%, nCD64 index, and CRP. (**D**) Decision curve analysis comparing the clinical net benefit of the combined models across a range of threshold probabilities. (**E**) Calibration curve of the optimized three-marker model generated using bootstrap resampling. (**F**) Nomogram based on the optimized three-marker model for estimating the probability of viral infection. AUC, area under the receiver operating characteristic curve; CRP, C-reactive protein; nCD64 index, neutrophil CD64 index.

ROC analysis was then used to evaluate the ability of individual biomarkers and combined models to discriminate viral from bacterial infection (Figure 4A–C). Among individual biomarkers, CRP yielded the highest AUC (0.8323, 95% CI: 0.7668–0.8979), while CD169% also showed good performance with an AUC of 0.8126 (95% CI: 0.7520–0.8733). At the optimal cutoff of CD169% > 43.15%, specificity for identifying viral infection reached 92.54%. The AUCs of HLA-DR% and the nCD64 index were 0.7707 and 0.6730, respectively (Table 4).

Among combined models, the initial three-marker flow cytometry model achieved an AUC of 0.8554 (95% CI: 0.7971–0.9138). Adding CRP improved the AUC of the four-marker model to 0.8946 (95% CI: 0.8461–0.9430), representing a significant increase compared with the initial three-marker model (DeLong test, Z = −2.2865, *p* = 0.0222). The optimized three-marker model combining CD169%, nCD64 index, and CRP achieved an AUC of 0.8947 (95% CI: 0.8463–0.9432), essentially identical to that of the four-marker model, with no significant difference between them. After internal validation with 1000 bootstrap resamples, the optimism-corrected AUC remained 0.8882. At a predicted probability cutoff of 0.6306, the optimized model showed a sensitivity of 81.25% and a specificity of 80.70% for identifying viral infection (Table 5). DCA indicated that this model provided greater net benefit than “treat-all” or “treat-none” strategies across a broad range of threshold probabilities (Figure 4D), and the calibration curve showed good agreement between predicted and observed probabilities (Figure 4E). A nomogram based on the optimized three-marker model is shown in Figure 4F.

### 3.5. Diagnostic Utility of the Combined Model in Mixed Infection

#### 3.5.1. Mixed Infection Versus Bacterial Infection

**Figure 5:**
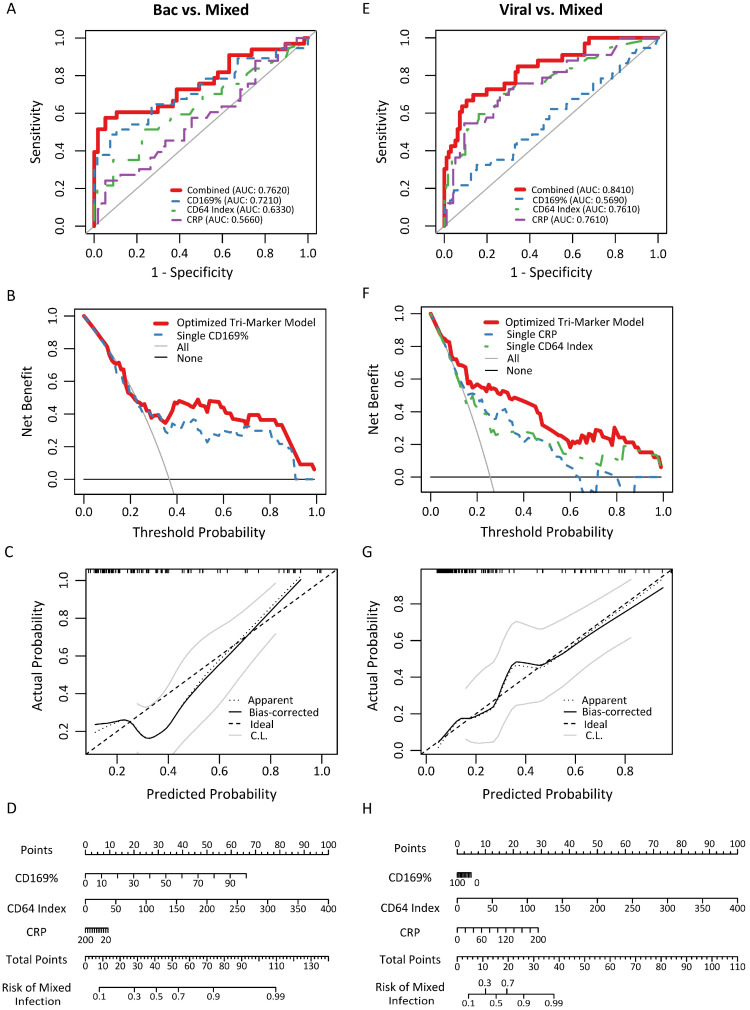
Diagnostic performance and clinical utility of combined models for identifying mixed infection. Mixed infection was coded as the positive outcome. (**A**) ROC curves for differentiating mixed infection from bacterial infection using CD169%, the nCD64 index, CRP, and the combined model. (**B**) Decision curve analysis for the model differentiating mixed infection from bacterial infection. (**C**) Calibration curve for the model differentiating mixed infection from bacterial infection. (**D**) Nomogram for estimating the probability of mixed infection in the comparison with bacterial infection. (**E**) ROC curves for differentiating mixed infection from viral infection using CD169%, the nCD64 index, CRP, and the combined model. (**F**) Decision curve analysis for the model differentiating mixed infection from viral infection. (**G**) Calibration curve for the model differentiating mixed infection from viral infection. (**H**) Nomogram for estimating the probability of mixed infection in the comparison with viral infection. The combined models were constructed using logistic regression. AUC, area under the ROC curve; CRP, C-reactive protein; nCD64 index, neutrophil CD64 index.

For discrimination between mixed infection and isolated bacterial infection, mixed infection was defined as the positive outcome. Analysis was restricted to patients with complete biomarker data. Among individual biomarkers, CD169% showed moderate discriminatory ability, with an AUC of 0.7360 (95% CI: 0.6200–0.8520). In contrast, the nCD64 index and CRP showed limited performance, with AUCs of 0.6060 and 0.5660, respectively. The combined model incorporating CD169%, nCD64 index, and CRP achieved an AUC of 0.7618 (95% CI: 0.6485–0.8751) (Figure 5A, Table 6). Although this was numerically higher than CD169% alone, the difference was not statistically significant by DeLong testing (*p* = 0.3284). At the optimal predicted probability cutoff of 0.4860, the model showed a sensitivity of 57.58% and a specificity of 94.74%.

Multivariable logistic regression indicated that CD169% was the principal independent predictor in this comparison, whereas the nCD64 index showed only a borderline association and CRP was not independently predictive (Supplementary Table S4). In patients with a bacterial inflammatory phenotype, increased CD169% may therefore serve as a useful clue to concomitant viral infection. DCA showed that the combined model provided greater net benefit than CD169% alone across part of the threshold range and outperformed both “treat-all” and “treat-none” strategies (Figure 5B). The calibration curve showed good agreement between predicted and observed probabilities (Figure 5C). A nomogram for differentiating mixed infection from isolated bacterial infection is shown in Figure 5D.

#### 3.5.2. Mixed Infection Versus Viral Infection

For discrimination between mixed infection and isolated viral infection, mixed infection was defined as the positive outcome and analysis was again restricted to complete cases. Among individual biomarkers, CRP and the nCD64 index showed moderate discriminatory performance, with AUCs of 0.7610 (95% CI: 0.6630–0.8600) and 0.7410 (95% CI: 0.6340–0.8480), respectively. CD169% showed limited discriminatory ability, with an AUC of 0.5710 (95% CI: 0.4500–0.6910), and was not statistically significant (*p* = 0.2276) (Figure 5E, Table 7). The combined model incorporating CD169%, nCD64 index, and CRP achieved an AUC of 0.8406 (95% CI: 0.7594–0.9218, *p* < 0.001). At the optimal predicted probability cutoff of 0.2826, the model showed a sensitivity of 66.67% and a specificity of 89.58%.

Although the combined model yielded a numerically higher AUC than either CRP or the nCD64 index alone, the improvement did not reach statistical significance compared with the nCD64 index (DeLong test, *p* = 0.0909) or CRP (*p* = 0.0611). Multivariable logistic regression showed that the nCD64 index and CRP were the main independent predictors in this setting, whereas CD169% did not retain independent value (Supplementary Table S4). Even without a statistically significant AUC increase over the strongest individual markers, DCA suggested greater clinical net benefit for the combined model than for single biomarkers or “treat-all”/“treat-none” strategies across a broad range of threshold probabilities (Figure 5F). Calibration also appeared acceptable (Figure 5G). A corresponding nomogram is shown in Figure 5H.

### 3.6. Dynamic Changes in Core Biomarkers During Treatment

**Figure 6:**
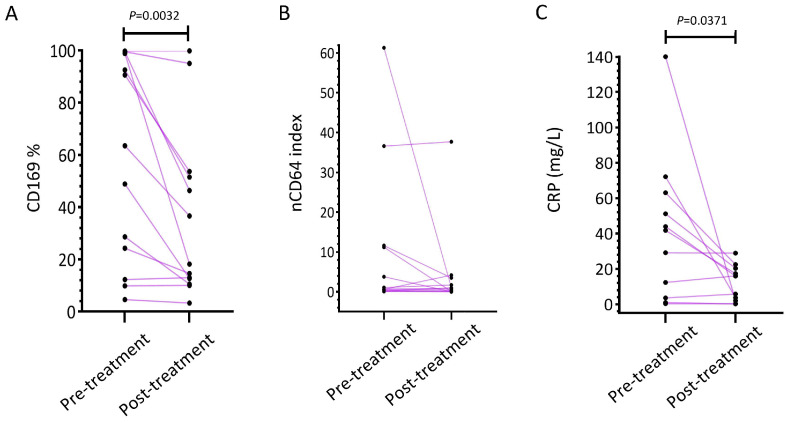

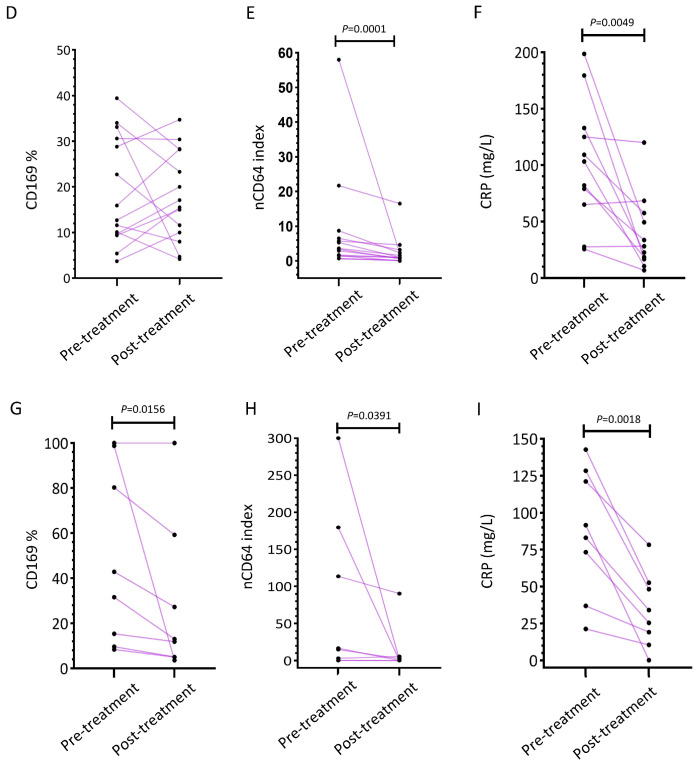
Dynamic changes in immune markers and CRP after treatment. Paired samples were obtained from 13 patients with viral infection, 14 patients with bacterial infection, and 8 patients with mixed infection. Follow-up samples were collected 3–5 days after treatment initiation. Changes in CD169%, the nCD64 index, and CRP are shown for viral infection (**A**–**C**), bacterial infection (**D**–**F**), and mixed infection (**G**–**I**). Each line represents one patient. Paired comparisons were performed using the Wilcoxon signed-rank test. CRP, C-reactive protein; nCD64 index, neutrophil CD64 index.

We next performed longitudinal monitoring to explore whether these biomarkers changed with treatment response. Thirty-five patients with complete paired follow-up data were included, comprising 13 with viral infection, 14 with bacterial infection, and 8 with mixed infection. Biomarkers were assessed at enrollment during the acute phase and again during recovery after clinically indicated anti-infective treatment (Figure 6).

In the viral infection group, CD169% decreased significantly during recovery (*p* = 0.0032; Figure 6A), and CRP also declined (*p* = 0.0371; Figure 6C). The nCD64 index remained low and did not change significantly (Figure 6B). In the bacterial infection group, both the nCD64 index and CRP decreased significantly after treatment (*p* = 0.0001 and *p* = 0.0049, respectively; Figure 6E,F), whereas CD169% remained relatively stable (Figure 6D). In the mixed infection group, CD169% (*p* = 0.0156; Figure 6G), the nCD64 index (*p* = 0.0391; Figure 6H), and CRP (*p* = 0.0018; Figure 6I) all declined during recovery. These paired data suggest that CD169% and the nCD64 index may track recovery along two different immune-response axes, one viral and one bacterial.

## 4. Discussion

Distinguishing viral, bacterial, and mixed respiratory infections early remains difficult in routine care [24]. Host-response biomarkers have attracted increasing interest because they may provide clinically useful information before pathogen-directed testing is finalized [11,25]. CD169, or Siglec-1, is regulated by type I interferon signaling and is rapidly upregulated on monocytes during viral infection [14,16]. Increased CD169 expression has been reported in influenza, respiratory syncytial virus infection, SARS-CoV-2 infection, and other acute viral illnesses, consistent with its role in antiviral immune activation [14,26,27,28]. Our results fit well with this biology. CD169% was higher in viral infection and remained the strongest variable for distinguishing mixed infection from bacterial infection. This suggests that a viral signal can still be detected even when the overall presentation is dominated by bacterial inflammatory features.

Neutrophil CD64 is minimally expressed on resting neutrophils but increases rapidly in response to bacterial infection, systemic inflammation, and sepsis [18,29,30,31]. Prior studies, including meta-analyses, have shown that neutrophil CD64 is useful for identifying bacterial infection and sepsis [19,29,30]. In our cohort, the nCD64 index was elevated in bacterial infection and in mixed infection, and it remained informative when mixed infection was compared with viral infection. Clinically, this is relevant because bacterial co-infection is often the key question in patients who initially appear to have a viral illness. CRP, although nonspecific, also performed well in our study, particularly when interpreted alongside cellular immune markers. CRP does not identify pathogen class on its own, but it reflects the intensity of the inflammatory response and is widely available in routine practice [32,33,34]. In our data, its main value was as a complementary variable rather than a stand-alone etiological marker.

HLA-DR% was lower in bacterial and mixed infection, consistent with impaired monocyte antigen presentation or infection-associated immune dysregulation [20,21]. However, once CRP was included in the multivariable model, HLA-DR% no longer contributed meaningful independent discriminatory value. In this setting, HLA-DR% may be more relevant to immune status, disease severity, or prognosis than to frontline etiological classification. This interpretation is consistent with the broader literature on monocyte HLA-DR in sepsis and immune suppression [20,21]. Taken together, the three retained markers were biologically complementary: CD169% tracked interferon-related antiviral activation, the nCD64 index reflected neutrophil activation associated with bacterial infection, and CRP captured the broader inflammatory response. This likely explains why the combined model performed better than the individual markers alone.

Our results are consistent with the broader move in infection diagnostics toward integrated host-response approaches [11,35]. Several existing assays follow the same general principle by combining a marker of viral immune activation with one associated with bacterial or inflammatory activity [36,37]. For example, FebriDx combines MxA and CRP [38,39], whereas MeMed BV integrates TRAIL, IP-10, and CRP into a composite host-protein score [36,40,41]. The model developed in our study follows a similar logic, but differs in several practical aspects. First, CD169% and the nCD64 index are cell-surface immune markers measured directly by multiparameter flow cytometry, allowing for simultaneous assessment of monocyte and neutrophil activation states from the same sample [12,13]. Second, we specifically evaluated mixed infection as a separate clinical category rather than collapsing it into either viral or bacterial infection. Third, we included paired follow-up data, which provided preliminary information on how these markers change during recovery [17]. At the same time, the feasibility of this approach depends on access to flow cytometry, standardized gating, and stable inter-laboratory performance. External validation across different centers and instruments will therefore be essential.

Mixed infection is particularly challenging to recognize early, and this was one of the main motivations for the present study. Prior work in influenza, COVID-19, and community-acquired pneumonia has shown that viral–bacterial co-infection is associated with greater illness severity, higher antibacterial use, and worse outcomes [42,43]. Even so, early mixed infection often resembles either bacterial infection or severe viral infection in routine clinical practice. For this reason, we analyzed mixed infection against bacterial infection and viral infection separately. This distinction matters clinically because the question asked by the treating physician depends on the initial presentation. In a patient who already appears to have bacterial infection, the key issue is whether there is also a viral component. In that comparison, CD169% carried most of the discriminatory information. In contrast, when the baseline presentation looks viral, the main clinical question is whether a bacterial co-infection is present and whether antibacterial therapy should be started or escalated. In that setting, the nCD64 index and CRP were more informative. This pattern is biologically plausible: the most useful marker is often the one that captures the response missing from the comparator group. One strength of our study is that mixed infection was treated as its own category rather than being absorbed into one of the monomicrobial groups. Even though the increase in AUC over the best individual marker was not statistically significant in every mixed-infection comparison, the combined models still showed potential clinical value in decision curve analysis.

Beyond baseline diagnosis, serial biomarker assessment may provide information that a single measurement cannot [17]. In our paired samples, CD169% decreased with recovery in viral infection, whereas the nCD64 index and CRP declined after treatment in bacterial infection. In mixed infection, all three markers fell during recovery, producing a pattern consistent with resolution of both the viral and bacterial components. These longitudinal findings were biologically coherent and suggest that CD169% and the nCD64 index may be useful for tracking recovery along two different immune axes, one viral and one bacterial [17]. This may be particularly relevant in mixed infection, where improvement depends on control of both components. However, the longitudinal analysis was exploratory, especially in the mixed-infection subgroup, and larger studies will be needed to determine whether the magnitude or speed of biomarker decline is associated with outcomes such as time to defervescence, duration of antimicrobial therapy, length of stay, ICU admission, or mortality.

From a clinical perspective, the combination of CD169%, nCD64 index, and CRP may be feasible in centers with routine flow cytometry access [12,13]. CRP is already part of standard laboratory testing in many hospitals, and CD169% and the nCD64 index can be obtained from peripheral blood using an established flow cytometric platform. The nomograms developed in this study were intended to make the combined models easier to apply at the bedside by translating biomarker values into individualized risk estimates [17]. In practice, such tools may be most useful when clinicians are uncertain whether the overall picture is viral, bacterial, or mixed, and whether antibacterial or antiviral therapy is warranted. It is important to emphasize, however, that this model is not a substitute for pathogen testing. Rather, it should be viewed as an adjunctive host-response tool that may support early clinical judgment while microbiological results are pending [11,44].

Several limitations should be noted. First, etiological adjudication incorporated the clinical course through discharge for hospitalized patients, whereas systematic longitudinal follow-up was unavailable for patients discharged directly from the emergency department. In addition, 32 patients with indeterminate etiology were excluded from the final analyses. Although this reduced reference-standard misclassification, it may have introduced selection bias by preferentially retaining patients with more definitive etiological evidence. CRP was also available to the adjudicating physicians as part of routine care; despite not being used as a standalone classification criterion, this may have introduced a degree of incorporation bias. Second, although specimens were collected before treatment at the index visit, the interval from symptom onset to sampling was not standardized. Temporal variation in CD169%, the nCD64 index, and CRP may therefore have contributed to biomarker heterogeneity. Third, the single-center design and geographically and seasonally restricted pathogen spectrum constrained the generalizability of our findings. Virological testing was restricted to SARS-CoV-2 and influenza A and B; therefore, infections or coinfections involving other respiratory viruses may have been missed, and the observed biomarker patterns and model performance may not extend to other viral etiologies. Likewise, reliance on culture-based criteria may have missed bacterial infections, particularly culture-negative cases. As the cohort included only adults, age-related differences in host immune responses may also limit extrapolation of these findings to children. Fourth, combined-model analyses were restricted to complete cases without imputation, and age differed among the etiological groups, potentially introducing selection bias and residual confounding. Finally, this panel was not systematically compared with other host-response biomarkers or commercial platforms [38,39,40,41], and the clinical effects of biomarker-guided implementation on antibiotic use and patient outcomes were not evaluated. Larger, multicenter, prospective studies incorporating standardized or serial sampling, external validation, and interventional assessment are warranted.

## 5. Conclusions

CD169%, the nCD64 index, and CRP reflect three complementary dimensions of the acute host response: interferon-related viral activation, neutrophil activation associated with bacterial infection, and systemic inflammatory burden [12,14,15,16,17,18,19,32,33,34]. A model integrating these markers showed good performance for differentiating viral from bacterial infection and also provided clinically relevant information in mixed infection. In patients with a bacterial inflammatory phenotype, CD169% may help identify a concomitant viral component, whereas in patients with a viral phenotype, the nCD64 index and CRP may help identify bacterial co-infection. With further validation across a broader spectrum of respiratory pathogens and in more diverse populations, this host-response-based strategy may become a useful adjunct for early etiological stratification and mixed-infection risk assessment [11,35,40,41].

## Figures and Tables

**Table 1:** Flow cytometry antibody panel for assessing CD169, HLA-DR, and neutrophil CD64 expression in whole blood.

Fluorochrome	Target Antigen	Clone	Manufacturer	Catalog No.	Volume per Test
FITC	CD16	NKP15	BD	335035	20 μL
PE	CD64	10.1	BD	652830	20 μL
PE-Cy7	CD19	SJ25C1	BD	341113	5 μL
APC	HLA-DR	L243	BD	665330	5 μL
APC-H7	CD14	MΦP9	BD	663492	5 μL
BV421	CD169	7-239	BD	742991	5 μL
V500-C	CD45	2D1	BD	662912	5 μL

**Table 2:** Baseline characteristics and laboratory parameters of healthy controls and patients stratified by infection type.

Variable	Healthy Controls (*n* = 50)	Viral Infection (*n* = 112)	Bacterial Infection (*n* = 67)	Mixed Infection (*n* = 37)	*p* Value
Sex, *n* (%)					0.11
Male	31 (62.0)	58 (51.8)	44 (65.7)	23 (62.2)	
Female	19 (38.0)	54 (48.2)	23 (34.3)	14 (37.8)	
Age, years	56 (46, 67)	62 (48, 73)	70 (55, 83)	69 (54, 77)	<0.0001
Laboratory parameters					
WBC, ×10^9^/L	6.04 (5.05, 6.67)	6.65 (4.80, 8.98)	10.25 (8.30, 11.84)	8.95 (5.03, 12.80)	<0.0001
NEU, %	55.35 (51.60, 58.95)	65.70 (55.80, 74.95)	81.00 (69.50, 87.90)	76.50 (61.10, 84.80)	<0.0001
LYM, %	34.95 (31.48, 37.90)	22.10 (12.65, 31.10)	9.80 (6.30, 16.70)	12.40 (4.70, 19.90)	<0.0001
MO, %	6.90 (6.30, 8.00)	8.40 (5.80, 10.00)	6.60 (4.20, 8.58)	6.30 (5.15, 8.80)	<0.0001
CRP, mg/L	NA	7.20 (1.30, 26.13)	52.40 (24.30, 93.65)	44.90 (13.25, 82.05)	<0.0001
CD169, %	17.75 (13.75, 32.63)	50.25 (24.53, 86.60)	17.20 (10.30, 33.10)	42.80 (17.20, 82.30)	<0.0001
HLA-DR, %	99.60 (98.88, 99.90)	99.20 (97.45, 99.70)	94.10 (79.40, 98.20)	98.90 (86.65, 99.60)	<0.0001
nCD64 index	0.10 (0.00, 0.50)	0.90 (0.20, 2.70)	2.70 (0.60, 6.50)	7.40 (1.35, 37.15)	<0.0001

Footnotes: Data are presented as median (interquartile range) for continuous variables and *n* (%) for categorical variables. *p* values indicate overall comparisons among groups. Continuous variables were compared using the Kruskal–Wallis test, and categorical variables were compared using the chi-square test or Fisher’s exact test, as appropriate. CRP was not measured in healthy controls; therefore, the *p* value for CRP was calculated among the three infected groups.

**Table 3:** Univariable logistic regression analysis of candidate biomarkers for differentiating viral infection from bacterial infection.

Variable	β Coefficient	*p* Value	OR	95% CI
CD169, %	0.0563	<0.001	1.0579	1.0390–1.0813
HLA-DR, %	0.1099	<0.001	1.1161	1.0685–1.1798
nCD64 index	−0.0341	0.0116	0.9664	0.9377–0.9900
CRP, mg/L	−0.0360	<0.001	0.9646	0.9506–0.9766

Footnotes: Univariable logistic regression was performed in patients with viral or bacterial infection. Viral infection was coded as the positive outcome, and bacterial infection was coded as the reference outcome. ORs were calculated per one-unit increase in each variable. For CD169 and HLA-DR, one unit corresponds to a one-percentage-point increase. OR, odds ratio; CI, confidence interval; CRP, C-reactive protein; nCD64 index, neutrophil CD64 index.

**Table 4:** Diagnostic performance of individual markers for discriminating viral infection from bacterial infection.

Marker	AUC	95% CI	*p* Value	Cutoff Value for Predicting Viral Infection	Sensitivity	Specificity
CD169, %	0.8126	0.7520–0.8733	<0.0001	>43.15	55.36%	92.54%
HLA-DR, %	0.7707	0.6971–0.8444	<0.0001	>98.25	70.54%	76.12%
nCD64 index	0.6730	0.5924–0.7537	0.0001	<1.35	64.29%	67.16%
CRP, mg/L	0.8323	0.7668–0.8979	<0.0001	<17.85	68.75%	87.72%

Footnotes: Viral infection was coded as the positive outcome, and bacterial infection was coded as the negative outcome in the ROC analysis. Cutoff values indicate thresholds for predicting viral infection and were determined using the Youden index. AUC, area under the receiver operating characteristic curve; CI, confidence interval; CRP, C-reactive protein; nCD64 index, neutrophil CD64 index.

**Table 5:** Diagnostic performance of combined models for differentiating viral infection from bacterial infection.

Model	AUC	95% CI	*p* Value	Cutoff Probability	Sensitivity	Specificity
Initial three-marker flow cytometry model: CD169% + nCD64 index + HLA-DR%	0.8554	0.7971–0.9138	<0.001	0.4814	88.54%	68.42%
Four-marker model: CD169% + nCD64 index + HLA-DR% + CRP	0.8946	0.8461–0.9430	<0.001	0.6303	81.25%	80.70%
Optimized three-marker model: CD169% + nCD64 index + CRP	0.8947	0.8463–0.9432	<0.001	0.6306	81.25%	80.70%

Footnotes: Viral infection was coded as the positive outcome. Cutoff probabilities were determined using the Youden index. Pairwise AUC comparisons were performed using the DeLong test. The four-marker model significantly outperformed the initial three-marker flow cytometry model (Z = −2.2865, *p* = 0.0222), while its AUC did not differ significantly from that of the optimized three-marker model. AUC, area under the receiver operating characteristic curve; CI, confidence interval; CRP, C-reactive protein; nCD64 index, neutrophil CD64 index.

**Table 6:** ROC analysis of individual markers and the combined model for differentiating mixed infection from bacterial infection.

Marker/Model	AUC	95% CI	*p* Value	Cutoff Value for Predicting Mixed Infection	Sensitivity	Specificity
CD169, %	0.7360	0.6200–0.8520	<0.001	>43.80	51.50%	94.70%
nCD64 index	0.6060	0.4770–0.7340	0.0973	>6.95	51.50%	73.70%
CRP, mg/L	0.5660	0.4400–0.6930	0.2992	<10.15	24.20%	94.70%
Combined model	0.7618	0.6485–0.8751	<0.001	>0.4860	57.58%	94.74%

Footnotes: Mixed infection was coded as the positive outcome, and bacterial infection was coded as the negative outcome in the ROC analysis. Analyses were performed in patients with complete data for the included markers. Cutoff values indicate thresholds for predicting mixed infection and were determined using the Youden index. The combined model included CD169%, nCD64 index, and CRP. Pairwise comparison of correlated AUCs was performed using the DeLong test; the AUC of the combined model was not significantly different from that of CD169% alone (*p* = 0.3284). AUC, area under the receiver operating characteristic curve; CI, confidence interval; CRP, C-reactive protein; nCD64 index, neutrophil CD64 index.

**Table 7:** ROC analysis of individual markers and the combined model for differentiating mixed infection from viral infection.

Marker/Model	AUC	95% CI	*p* Value	Cutoff Value for Predicting Mixed Infection	Sensitivity	Specificity
CD169, %	0.5710	0.4500–0.6910	0.2276	<20.80	33.30%	85.40%
nCD64 index	0.7410	0.6340–0.8480	<0.001	>2.35	69.70%	70.80%
CRP, mg/L	0.7610	0.6630–0.8600	<0.001	>42.85	54.50%	90.60%
Combined model	0.8406	0.7594–0.9218	<0.001	>0.2826	66.67%	89.58%

Footnotes: Mixed infection was coded as the positive outcome, and viral infection was coded as the negative outcome in the ROC analysis. Analyses were performed in patients with complete data for the included markers. Cutoff values indicate thresholds for predicting mixed infection and were determined using the Youden index. The combined model included CD169%, nCD64 index, and CRP. Pairwise comparisons of correlated AUCs were performed using the DeLong test; the AUC of the combined model was not significantly different from that of nCD64 index alone (*p* = 0.0909) or CRP alone (*p* = 0.0611). AUC, area under the receiver operating characteristic curve; CI, confidence interval; CRP, C-reactive protein; nCD64 index, neutrophil CD64 index.

## Data Availability

The original contributions presented in this study are included in the article/Supplementary Materials. Further inquiries can be directed to the corresponding authors.

## Supplementary Materials

The following supporting information can be downloaded at https://www.mdpi.com/article/10.3390/diagnostics16152416/s1, Supplementary Figure S1: Distribution of conventional inflammatory markers among healthy controls and patients with different infection types; Supplementary Table S1: Summary of pathogen infection data; Supplementary Table S2: Hospitalization information by infection group; Supplementary Tables S3 and S4: Multivariable logistic regression results.

## Abbreviations

AIC	Akaike information criterion
AUC	Area under the receiver operating characteristic curve
BD	Becton, Dickinson and Company
CI	Confidence interval
CRP	C-reactive protein
DCA	Decision curve analysis
EDTA	Ethylene Diamine Tetraacetic Acid
FSC	Forward scatter
HLA-DR	Human Leukocyte Antigen—DR
IQR	Interquartile range
MO%	Monocyte percentage
MFI	Mean fluorescence intensity
NEU%	Neutrophil percentage
LYM%	Lymphocyte percentage
nCD64 index	Neutrophil CD64 index
OR	Odds ratio
PCR	Polymerase chain reaction
ROC	Receiver operating characteristic
SSC	Side scatter
WBC	White blood cell count
